# Ergonomic Risk Assessment of Professional Dance Using Motion Capture with Ergonomic Evaluation by the Rapid Entire Body Assessment (REBA)

**DOI:** 10.3390/s26010070

**Published:** 2025-12-22

**Authors:** Verena Fehringer, Christian Maurer-Grubinger, Fabian Holzgreve, Daniela Ohlendorf, Eileen M. Wanke

**Affiliations:** Institute of Occupational, Social and Environmental Medicine, Johann Wolfgang von Goethe University, Theodor-Stern Kai 7, Haus 9a, 60590 Frankfurt am Main, Germany; verena.dickel@web.de (V.F.); christian.maurer.cm@gmail.com (C.M.-G.); holzgreve@med.uni-frankfurt.de (F.H.); ohlendorf@med.uni-frankfurt.de (D.O.)

**Keywords:** ergonomics, inertial motion capture, kinematic analysis, musculoskeletal disorders, risk assessment, professional dance

## Abstract

The aim of the present study was to assess physical demands in professional dance during daily training routine using kinematic data and to categorize it ergonomically using the Rapid Entire Body Assessment (REBA) tool. The three phases of daily classical ballet training of n = 28 professional dancers (16f/12m) were recorded with the inertial motion capture system MVN Link (Xsens, Netherlands), extracted and analyzed by MATLAB; subsequently, the ergonomic risk was determined. Female dancers trained significantly longer in the high-risk range than their male colleagues (f: 94%; m: 89%; *p* < 0.001). During the entire training, the female and male dancers had a mean REBA score of 6.31 and 6.03 resp., with phase 3 tending to have lower REBA values but an increased likelihood of injury due to fatigue and ground reaction forces. It can be recommended that the daily training should be critically examined and adjusted to anthropometric characteristics and the integration of regeneration phases, cardiopulmonary components, and targeted strength training programs to relieve vulnerable structures, as substantiated in the main text and should not exaggerate the main conclusions.

## 1. Introduction

Professional dance in the 21st century is associated with very high strain on the musculoskeletal system. Despite the fact that professional dance belongs to the field of performing arts, dancers in this context are investigated as employed workers who have very few work tools at their disposal—their work tool is their own body. The basis for practising the profession of a professional dancer is an appropriate specific technique. The significance of dance as an art form is therefore secondary in the context of this study.

Vocational ballet training begins predominantly in the prepubertal years and continues throughout the entire course of active professional practice [1,2].

The daily classical training forms the basis for rehearsals and performances. The training follows a traditional, characteristic methodological and didactic structure [3]. On the one hand, this serves to maintain and further develop dance technique and, on the other, is intended to prevent injuries and prepare for subsequent activities such as rehearsals and performances. In cardiopulmonary terms, the interval-style training is structured primarily at the end of barre training and, in the final, jump-focussed phase, in the submaximal intensity range with lactate values in the anaerobic intensity range [3].

Elements of movement that can cause musculoskeletal complaints and disorders, such as a high static component, rotational movements, repetitive movements, carrying and lifting heavy loads and adopting end-degree joint positions, high force effects or high ground reaction forces after jump landings [4], are among the characteristics of daily training and rehearsal content in professional dance [5,6]. The results of these high strains can be chronic injuries and overuse injuries as well as acute injuries that occur during the work process [7]; every second dancer suffers an acute injury at least once in the course of a year, with chronic, overuse injuries varying between three and six times more frequently [7].

With regard to injury patterns, age-, gender-, and style-specific differences must be taken into account [8,9,10]. In addition, temporal and content-related correlations can be observed, with injuries resulting from jumps at the end of training being among the movement elements that most frequently lead to acute injuries in this phase of activity [7].

For professional dance, there have been no analyses, to date, which classify the characteristic activities and movement content qualitatively and quantitatively, which would allow the ergonomic risk to be determined through professional practice. Suitable methods include observational assessment tools such as the Rapid Upper Limb Assessment (RULA) [11] or Rapid Entire Body Assessment (REBA) [12]. RULA was designed as an ergonomic assessment tool for analysing the risk of work-related upper limb disorders, and it identifies the need for action, while REBA similarly assesses the entire body [11,12]. With regard to REBA, which is also relevant to dance, this has already been widely implemented in the manufacturing industry, agriculture, forestry and fisheries, health and social services, transport and storage, and construction [12,13]. It is possible to combine the aforementioned observational assessment methods, which are based on static momentary measurements, with Inertial Measurement Units (IMUs) [14]. This combination provides a more precise analysis of body postures and movements in the working environment by continuously recording data over a defined period. The resulting ability to record complex or repetitive motion sequences in real time with angle, acceleration and rotation data enables an objective assessment. This combination has so far been realized with RULA [15] and has already been applied several times to the occupational group of dentists with different questions [16,17,18,19,20], and also to industrial workers [21] and physical therapists [22]. Maurer-Grubinger et al. [15] have proposed a method to automate the calculation of RULA with the combination of IMU sensors and custom-built MATLAB (Version 2024a) routines.

For REBA, this combination has, so far, only been used with regard to work-related musculoskeletal disorders (WMSDs) during bed-to-wheelchair and wheelchair-to-commode transfers [23] but has not been applied to an occupational setting for a selected occupational group. The use of inertial sensors to analyze work ergonomics has, thus, provided promising data in recent years and offers many advantages. However, it has not yet been implemented in the workplace [24]. Hita-Gutierrez et al. [12] reported that REBA is a useful method for identifying the forced postures workers must assume in their daily activities. The combination of REBA with Xsens MVN link offers, for the first time, the possibility not only to determine the ergonomic risk in comparison with other occupational groups, but also to improve preventive measures or the recognition of work-specific stresses in professional dance.

Therefore, the aim of the present study was, compared to existing biomechanical approaches [25], not only to record qualitatively and quantitatively, for the first time, the classical dance training associated with typical, complex movement elements of different sequences but to additionally carry out an ergonomic risk assessment of the entire body with REBA using a motion capture system [26]. In particular, gender-specific differences will be analyzed here. In addition, the ground reaction forces during training were identified. The results could help to question traditional training content and its methodological and didactic processes and be used to develop initial ideas for preventive concepts. At the same time, a database was to be created that would improve the recognition of work-related musculoskeletal complaints or occupational illnesses [27].

## 2. Materials and Methods

### 2.1. Subjects

A total of n = 28 (16 female; 12 male) professional dancers aged between 22 and 38 years (females: 30.8 ± 3.9; males: 29 ± 6.5), from the German ballet companies of the Oldenburg State Theatre and the Kiel Theatre, voluntarily participated in the present study. The average female body height was 162.9 ± 5.8 cm and 174.6 ± 6.5 cm for the males; the average female body weight was 50.1 ± 3.5 kg and 75.4 ± 6.9 kg for the males. All dancers were included who had a full-time employment contract in the respective company at the time of the study measurement and were subjectively physically fully fit and, thus, free of restrictive physical complaints or pain. All dancers completed the classical training included in the analyses. The traditional classical dance training we discuss has hardly changed in almost 350 years. Only the composition of the core movement elements of each exercise changes. The results of classical training sessions are therefore expected to be similar. This means that our findings regarding this form of training can be generalised. The strengths and weaknesses have been outlined in the limitations section.

Exclusion criteria were having a guest status (production contract), injuries of the musculoskeletal system that were associated with a restriction of movement or exertion in the daily routine, or other acute illnesses (e.g., infections). Written consent was obtained from the participants before the start of the study. This study was conducted in accordance with the Declaration of Helsinki and was approved by the medical ethics board for research involving human subjects of the Goethe University (Nr. E 115/23) in Frankfurt am Main, Germany.

### 2.2. Measurement: Xsens MVN Link

**Figure 1:**
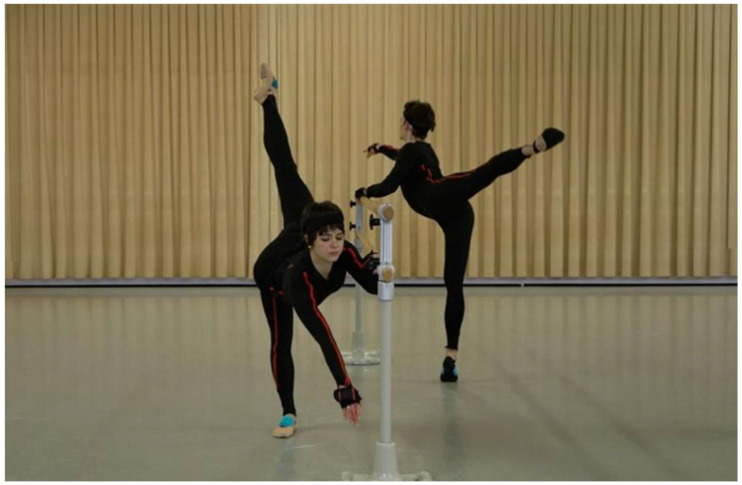
Special suit with the integrated 17 sensors during training at the barre (so-called phase 1).

The MVN Link person-bound inertial motion capture (IMC) system from Xsens (Enschede, The Netherlands) was used to record the complex movement sequences during professional dance training. The system consisted of 17 inertial measurement units (IMU) and a body pack with a battery. To ensure that the sensors could be attached to the head, torso, upper and lower arms, wrists, upper and lower legs, and feet without slipping, according to the manufacturer, each participant wore a special suit (Figure 1) in which they were connected to one another via cables. Each sensor contained a linear 3D accelerometer, a gyroscope, a barometer and a magnetometer.

The sampling rate was 240 Hz with the manufacturer’s measurement error of ±1% [28]. The system interpolated 22 joints with three dimensions and the position and orientation data of 23 segments. Compared to optical motion capture, which is considered the gold standard, the inertial motion capture system provides good to very good measurement values, especially for the frontal and sagittal planes of ±2° and slightly larger deviations of ±5° in the transverse plane [29,30]. When comparing the Xsens MVN Link system with the Optotrak system, a mean square error of 2.8° for long, complex movement tasks and an error of 1.2° (*p* ≤ 0.001) for short, functional movements have been obtained [31]. Fang et al. [32] described differences of 4° across all degrees of freedom of humerothoracic movement, although higher accuracies were reported at the elbow joint of 2°, for the glenohumeral joint of >6° and in the scapulothoracic joint of >10° in the protraction-retraction and anterior–posterior tilt directions. The ground reaction forces were estimated based on the second derivative of the vertical pelvic position. The forces (in units of body weight) were calculated as:

$$a=\frac{d^{2}z}{dt^{2}}$$

With *z* being the vertical movement of the pelvic centre. The major limitation of this approach is the underestimation of the ground reaction forces, especially during landing [33].

### 2.3. Rapid Entire Body Assessment (REBA)

**Figure 2:**
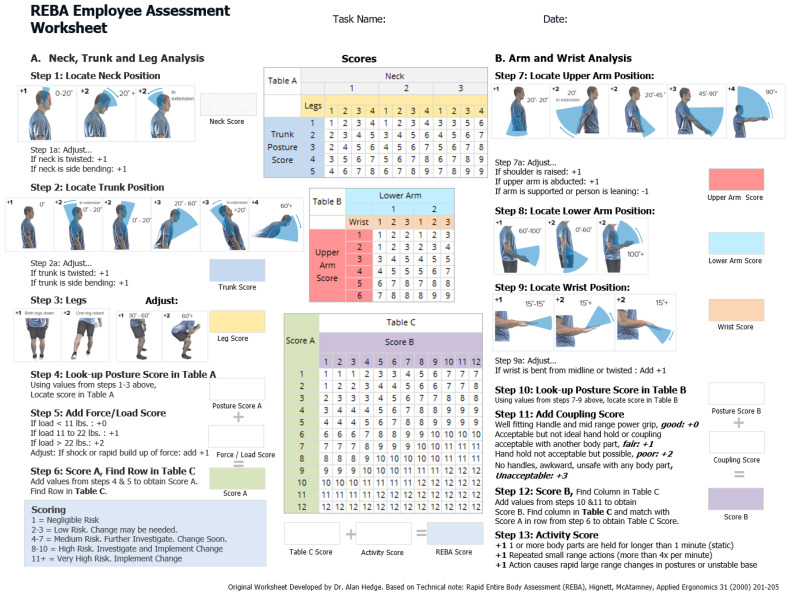
REBA—Employee Assessment Worksheet [34,35].

**Figure 3:**
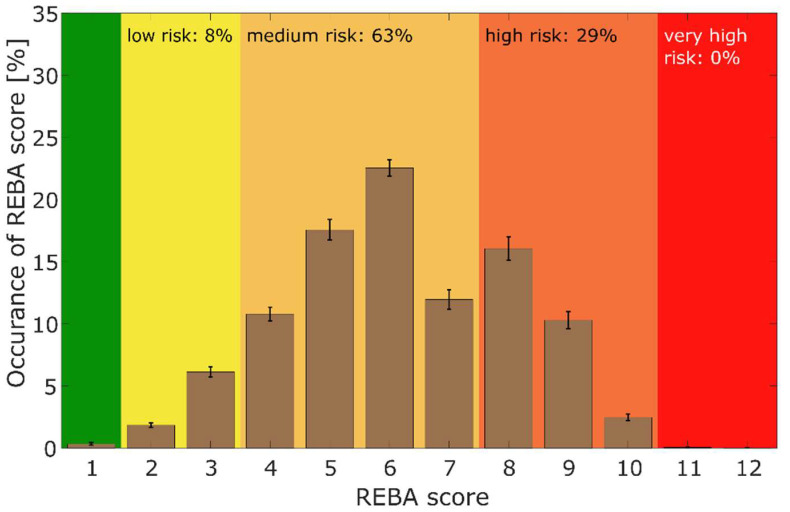
Total REBA score during professional training with regard to the distribution (occurrence) of the scores [%] (n = 28). Color coding: green: score 1, yellow: score 2–3, orange: score 4–7, apricot: score 8–10, red: score ≥ 11.

The Rapid Entire Body Assessment (REBA) was developed by Hignett & McAtamney and is used for the rapid assessment of ergonomic risk by means of an observational motion analysis by an observer (Figure 2) [26,34]. It divides the body into separately coded parts (for example, the trunk, legs, neck, upper and lower arms) and assigns a score to muscle activity in static, dynamic, rapidly changing, or unstable postures [20,23]. The assessment is carried out using a point scale based on various factors. The position of the joints (e.g., the hips, knees, or spine), the posture of the body, the strain on muscles and joints, the frequency of movements and their duration, as well as the resulting ground reaction forces during the jumps, estimated via the acceleration of the pelvic markers, are taken into account. The REBA scores range from 0 to 15 [29] (Figure 3). The higher the score, the greater the risk of developing work-related musculoskeletal disorders.

The ergonomic risk is then determined from the overall REBA score and represents the need for action according to the final score that is listed in Table 1.

### 2.4. Conception of Classic Training

The classical training was chosen because it provides an important basis for the technical preparation of the body and for maintaining technical dance competence in everyday dancing, even if contemporary or neoclassical choreography follows later in the day. The compulsory 75–90-min training programme, including breaks, begins at the barre and continues in the centre of the studio. The pure load duration accounts for about 50 per cent of the total training duration [3]. The elements to be practised can be integrated into a varying step sequence on a daily basis in order to bring as much variety as possible into the daily routine [6]. The structure and step content (but not the connections between the individual steps) of classic training follow a historically developed methodology with different sections that build on each other (phase 1: barre training, phase 2: centre work, up to the jumps, phase 3: jumps) [3,6,36]. The intensity of the load increases as the training progresses; initially, this is static, supported and with long movement elements (>2 min) with short pauses that progress into sprint-like, very dynamic movement patterns with very short sequences and long pauses [3,6,36]. The more dynamic and expansive the movement elements become, the fewer dancers perform the exercise at the same time. The movement sequences are performed with the right and left leg as the free leg. It is customary to include a pause between the right and left free leg side in order to teach the subsequent step combinations. Individual movement sequences in free space (e.g., adagio, pirouettes, jump combinations, possibly working with pointe shoes) are sometimes repeated (see Table 2). Due to the gender-specific movement elements in classical training, the training may differ slightly between male and female dancers.

Table 2 shows the training content carried out for the measurements and the proportion of time that it lasted. The total training time in this study was 80 min. Repetitions of the movement sequences in free space were taken into account for the pirouettes, the ‘petit allegro’, ‘batterie’, ‘grand allegro’, and ‘allegro en manege (m)’ or ‘pirouettes en manege (f)’. A classical training programme was developed for the study that corresponded to the technical dance level of professional dancers. The content was determined by the director of the Oldenburg State Theatre company, Antoine Jully, in cooperation with the study management. As usual, the specific step sequences were taught by the training instructors directly before the upcoming movement sequence. In order to guarantee identical conditions in terms of speed, the live piano accompaniment was replaced by a CD recording. The time slots were determined in advance to avoid waiting and ensure the training could be carried out as realistically as possible. The warm-up before the training session was carried out individually.

### 2.5. Study Procedure

The study was conducted in the ballet studios of the theatre in Kiel and the state theatre in Oldenburg using an identical set-up with the respective ballet ensembles (Kiel: n = 15; Oldenburg: n = 13). On the day of the study, the Xsens MVN Link measurement system was calibrated for each dancer with a neutral posture and a short walking sequence, as specified by the manufacturer. The recording was only started if the calibration quality was indicated as “good”. The recordings were then started in parallel with the start of training. At the same time, the entire measurement was filmed with an iPad Air or a Samsung tablet to track any deviations and assign the respective movements. Measurements were recorded using the MVN Analyze 2024.2.1 software from Movella Inc. (Enschede, The Netherlands) and exported for analysis. In addition, video recordings of all movement sequences were made in order to be able to synchronise the measurements and film recordings later on. The iPad videos were captured to have a clear visualisation of the full scene, including the surroundings, without any digitalization. This footage was used in case of abnormal movement capturing to verify if these abnormalities are based on extreme movements or on malfunctions of the capturing device. Only pure movement sequences without waiting times or learning phases were documented; this resulted in n = 19 (female) or n = 16 (male) individual measurements per participant, depending on gender. The higher number of measurements for the female dancers was due to the pointe shoe work; in all recordings, 2 dancers were measured simultaneously.

### 2.6. Methodological Evaluation

Table 3 shows the specific adjustment parameters for calculating the REBA score based on the concept of Hignett & McAtamney [34].

### 2.7. Statistical Evaluation

After high-quality reprocessing in the Xsens MVN Link, the data were exported, and the REBA scores for each measurement time point were calculated in MATLAB (2024 a) (REBA source code can be found in the Supplementary Material). The relative REBA scores were determined using the frequencies of the REBA scores occurring in each case. First, the absolute frequencies were calculated for all movement sequences and then normalised to the total length of the respective movement sequences. The mean REBA score was derived from the relative REBA scores. For the load, the frequency was determined between 0 and 5 times the bodyweight (BW) in 0.5 graduations of BW. The mean load was calculated from this frequency distribution. The statistical analysis was also performed in MATLAB. The data were tested for normal distribution using the Kolmogorov–Smirnov test. As the data were not normally distributed, the non-parametric Wilcoxon–Mann–Whitney U test (MWU) was used to compare the genders and the Kruskal–Wallis test (KW) was used for comparisons with more than two groups. For multiple tests, such as the REBA detailed analysis, a Bonferroni–Holm correction was applied to counteract alpha error accumulation. The Bonferroni–Holm correction was applied to the individual comparisons (between the different phase conditions and between the different movement sequencies). A Cliff’s delta δ calculation was used to calculate the effect size within the non-parametric tests. For the interpretation of the effect size, we used the following rule: An effect size of |δ|< 0.33 was considered as a small effect; 0.33 < |δ|< 0.5 a medium effect size; |δ|> 0.5 a large effect size. The significance level was set at 5%.

## 3. Results

### 3.1. Total REBA Score and Load

Figure 3 contains the total REBA score of all the dancers during training. In the overall assessment of the ergonomic risk based on the REBA score, 63% of the daily training time was spent in the medium risk range, whilst 29% was in the high risk range, thus meaning that 92% of the training took place in the medium to high risk range.

**Figure 4:**
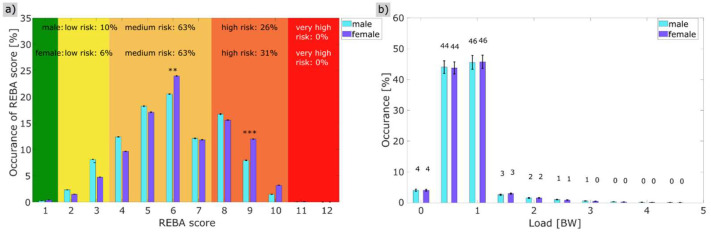
(**a**,**b**): (**a**) Percentage of the gender-specific REBA total score during professional training (n = 28); (**b**) gender-specific distribution of the load (body weight (BW)) with regard to occurrence in % (n = 28). Color coding = green: score 1, yellow: score 2–3, orange: score 4–7, apricot: score 8–10, red: score ≥ 11 (n = 28). Significance below 0.0042 are labeled **, significance below 0.001 are labeled as ***.

Figure 4a shows the gender-specific comparisons in the overall training analysis. The REBA score for male dancers was 63% in the medium risk range (REBA 4–7) and 26% in the high risk range (REBA 8–10). At 63% (REBA 4–7), the female dancers showed a comparable distribution in the medium risk range, although there was a higher proportion (31%) in the high risk range (REBA 8–10) than the male dancers. However, when the high and medium risk ranges are combined, it can be seen that 94% of the female dancers and 89% of the male dancers were in the higher medium risk range or above during training. The mean REBA score for female dancers was 6.31 [6.23; 6.50] and that of male dancers was 6.03 [5.72; 6.18] effect size = 0.36, thus, the female dancers had a significantly higher REBA load than the male dancers (Wilcoxon test: MWU = −3.34 (*p* < 0.001)) but with a low effect size, meaning there is no relevant difference between male and female dancer The detailed analysis showed differences in REBA scores 6 and 9, with the dancers having a significantly higher proportion on REBA score 6: MWU = −2.989 (*p* = 0.003) and a lower proportion on REBA score 9: MWU = −3.343 (*p* = 0.001) (Figure 4a, Table 4).

**Figure 5:**
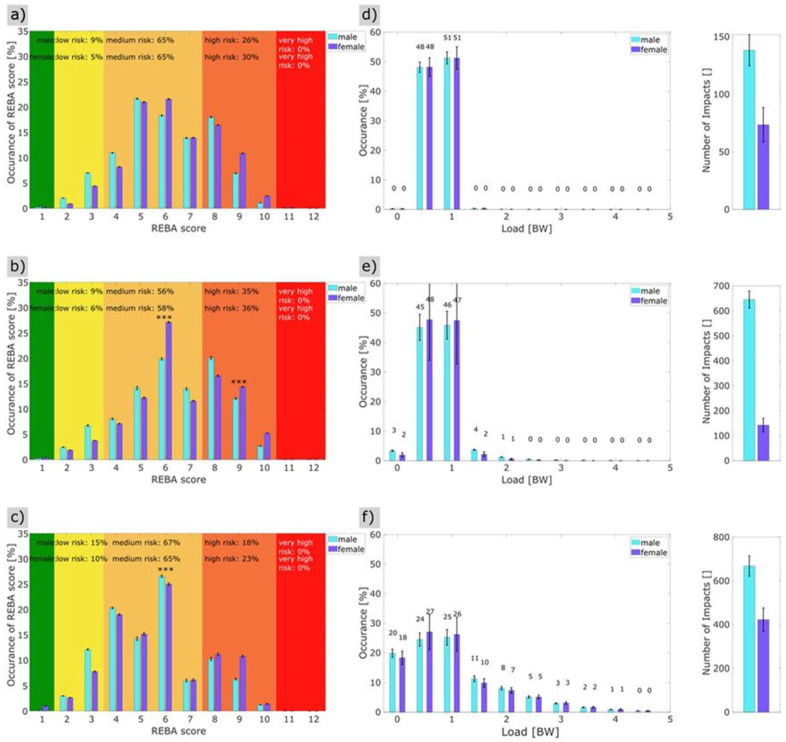
(**a**–**f**): Time proportions of the gender-specific REBA total score during the phases of training and relative distribution of the load (body weight (BW)) (n = 28). (**a**) REBA score phase 1, (**b**) REBA score phase 2, (**c**) REBA score phase 3, (**d**) load phase 1, (**e**) load phase 2, (**f**) load phase 3. Color coding: green: score 1, yellow: score 2–3, orange: score 4–7, apricot: score 8–10, red: score ≥11. Significance below 0.001 are labeled as ***.

The relative analysis of the load over the entire effective training time showed no significant gender-specific differences. The mean load was 1.31 BW for the male dancers and 1.31 BW for the female dancers, MWU = 0.99 (*p* = 0.32) (Figure 5b).

### 3.2. Phase-Specific REBA Score and Load Taking Gender into Account

#### 3.2.1. Phase 1 (Barre)

The analysis of the ergonomic risk during the first phase of training at the barre with supported movement elements showed a significant gender-specific difference (Figure 5a,d). The mean REBA score was 6.34 for the female dancers and 6.06 for the males, with a Wilcoxon–Mann–Whitney U test (MWU) of 2.25 (*p* = 0.024); this difference was also reflected in a lower proportion of male dancers in the high risk range (26%) than the females (30%). No significant gender-specific differences (*p* = 0.83) were found with regard to the mean load compared to the physical load (Figure 5d).

#### 3.2.2. Phase 2 (Centre Work, Jumps Excluded)

In phase 1 of the training, there were no gender-specific differences on average (*p* = 0.3). However, the detailed analysis showed a significant difference between the genders in REBA score 6 and 9 (REBA score 6: MWU = 3.857 (*p* < 0.001) and REBA score 9: MWU = 3.434 (*p* < 0.001) (Table 5). The dancers spent 55% of their time in the medium risk range and 36% in the high risk range (Figure 5b). For the female dancers, 58% of the load was in the medium risk range and 36% in the high risk range. The ground reaction forces for the female dancers were, on average, half their body weight for 48% of the total time of phase 2 and, on average, one times their body weight for 47% of the time of phase 2. For the dancers, these were, on average, half their body weight for 45% of the time and their full body weight for 46% of the time (Figure 5e). The mean values of the acting ground reaction forces (load) differed significantly between the two genders. The mean load was (male; female) = (1.26; 1.28); Wilcoxon rank sum (MWU) = 4.37 (*p* < 0.001).

#### 3.2.3. Phase 3 (Jumps)

In the evaluation of ergonomic risk, the third phase of training showed 67% in the medium risk range and 16% in the high risk range for male dancers, and 67% in the medium risk range and 20% in the high risk range for female dancers. The gender-specific differences were not significant, on average (*p* = 0.08) (Figure 5c). However, the detailed analysis revealed a gender-specific difference in REBA score 6: MWU = −3.923 (*p* = 0.000) (Table 4) and, in the relative representation based on the REBA scores, additional burdens were found for female dancers in the high-risk area (REBA score 8: MWU = −1.137 (*p* = 0.255) and REBA score 9: MWU = 1.648 (*p* = 0.099) (Figure 5c, Table 4), however, these were not significant.

The ground reaction forces were relatively distributed among the female and male dancers in the range of between 0 (jumps, air phase, without their own body weight) and 3 times body weight in the pelvic area during landings after jumps. For 20% of the time in phase 3, the dancers were in the air (no ground reaction forces) and for 24% of the time in the range of half (0.5 times) their body weight, whilst for 25% of the time, the forces were in the range of one times their body weight, and for 24% of phase 3 time above that value. There was no significant difference in the mean load between the two sexes (*p* = 0.47). The flight phase accounted for 18% of the load, and in 27% the ground reaction forces were only measured in the reduced body weight during the preparation for the jump. In the third phase, 26% of the time the load was the simple body weight, and 22% of the time the load was greater than body weight, for example, during the push-off and landing phases. (Figure 5f).

**Figure 6:**
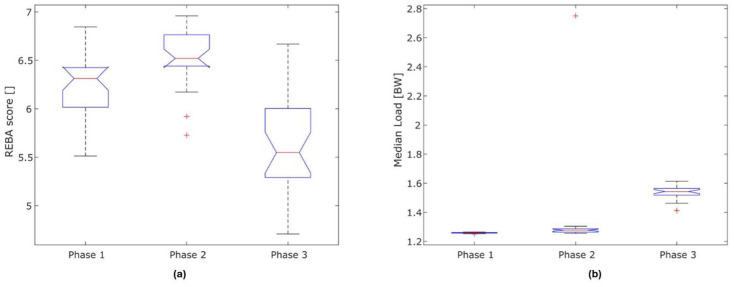
(**a**,**b**): Illustration of the phase-specific REBA score (n = 28) (**a**) and the load (**b**). Calculation of the REBA score of the individual movement sequences of the training, taking gender into account.

Figure 6a,b show the significant differences in the mean REBA scores and ground reaction forces in the three phases of training. The mean REBA score differed significantly for the three phases: (phase 1; phase 2; phase 3): (6.31; 6.52; 5.55) *p* < 0.001 (n = 28) (Kruskal–Wallis test). Table 6 shows the results of the paired analysis. Significant differences were also found for the mean load: phase 1 = 1.26 BW, phase 2 = 1.28 BW, phase 3 = 1.54 BW.

Table 6 and Table 7 show the gender-specific ergonomic risk of the training units categorised according to their corresponding phase, taking into account the subcomponents of each phase. The mean REBA score was also taken into account separately for each movement sequence according to gender, as well as the MWU (p). In phase 1 of the training, the female dancers with higher REBA scores were significantly more stressed (plié: MWU −3.17 (*p* < 0.002); adagio with battement fondu, ronds de jambe en l’air, developpé: MWU −3.1 (*p* < 0.002)). The gender-specific differences are also evident again in phase 2 of the training with regard to the additional load on the female dancers (port de bras and adagio: MWU −5.2 (*p* < 0.001)). The dynamic phase of the jumps (phase 3) showed no significant differences (jetés/glissades, jeté, assemblé and others: MWU −2.13 (*p* < 0.03)). However, there was an increase in the stress of the professional dancers.

## 4. Discussion

Professional ballet dancers are exposed to very high physical strain over many years [37,38]. To date, an ergonomic categorization of the movement sequences with regard to a specific risk for the development of work-related musculoskeletal disorders has not yet been carried out. Therefore, this study aimed to record the physical stresses experienced by professional dancers during their daily training using kinematic data, to analyze the stresses by using the Rapid Entire Body Assessment tool (REBA) and to classify them with regard to the ergonomic risk of developing musculoskeletal disorders. Building on this study, the next steps will be to use the created base to propose and develop specific preventive recommendations for action.

Training introduces the daily work routine and is supposed to be the best protection against injuries [3,39]. Based on the data collected, however, the present study showed that there already exists a high ergonomic risk during training, which, thus, justifies preventive measures. In the overall assessment, 63% of the pure training time was spent in the medium risk range and 29% in the high-risk range, thus meaning that a total of 92% of the net load duration took place in the medium to high-risk range. From an ergonomic perspective, the load, which can be classified as high, can be compared to sport physiological studies, which prove a considerable physical strain on dancers. This strain is closely linked to specific movement elements that are an integral part of training and are considered potential risk factors for the development of musculoskeletal disorders [4]. These elements include the high static components (e.g., holding an arabesque), rotational and torsional movements (e.g., port de bras, jumps, lifts), repetitive movements (e.g., jumps) and the adoption of terminal joint positions (e.g., grand plié) [4]. The lactate concentrations resulting from the cardiopulmonary stress experienced by dancers during training were in the anaerobic range, an observation that has been described several times (e.g., [40,41]).

The ergonomic load throughout the training program showed significant gender-specific differences, whereby the results are limited due to the size of the group studied or other aspects that we are not yet able to assess conclusively. Therefore, the results have to be discussed very carefully. The female ballet dancers had a significantly higher percentage of ergonomic stress in the high-risk range. In terms of the total time spent training at the barre (phase 1), the female participants completed 94% of their training in the medium to high-risk ranges, while for male participants, 89% of their training was within these ranges. The reasons for this could be due to the anthropometric differences [41,42,43,44,45] and the female dancers’ increased flexibility compared to the male dancers since both aspects involve further ergonomic risks and increased injury potential of the lower extremities [46]. Nevertheless, the actual reasons for the higher ergonomic risk must be clarified in further analysis.

While at the beginning of classical dance training static movement elements predominate on the floor, this changes in the course of the training leading up to big jump combinations [3].

Although a comparably high REBA score of 6–9, on average, could be determined in the first and second phases, phase 3 differed significantly from the other two since, in addition to all other aspects included in the REBA score, the ground reaction forces after landings were taken into consideration; these were recorded with the help of the pelvic sensor. However, it must be taken into account that the forces acting on the pelvis were not only estimated and already absorbed by the joints of the ankle, knee and skeletal structures, so that the significance in this respect was reduced [4,5]. If one considers the forces acting as ground reaction forces, these were still low at the beginning of the training and were also not gender-specific. However, as the dynamics of the movement content, in the form of jumping, rotation movements and lifts, characterizing the last phase of the training increased, differences in the genders became more apparent. Thus, it can not be excluded that the ground reaction forces reflect the real intensity of the training content [47].

Phase 2 of the training took place in the open space (center) and initiated the transition to the jumps. Here, the subgroups of the REBA score (low risk, medium risk and high risk) differed by gender as REBA values, whereas the mean REBA score did not differ significantly. This may be due to the increase in the dynamic component despite the static movement elements or to the generally higher flexibility of female dancers [46,48,49]. Further analyses are required here to determine the cause; however, it cannot be ruled out that this aspect may not be conclusively clarified.

Phase 3 of the training was characterized by the sprint-like, very dynamic movement patterns with many jumps. The ergonomic risk based on the REBA score was reduced in favor of the medium risk; however, the dancers still had components with a high REBA score that may be present due to the jump combinations. Due to the increasing dynamics, the number of end-degree joint positions was reduced since the high number and types of jumps meant that these could not be stably achieved; thus, this may be the cause of the lower REBA scores. Nevertheless, studies found that the risk of injury in this phase shortly before the end of training is increased anyhow due to the coordinative and cardiopulmonary demands in the anaerobic range and the fact that the first signs of fatigue can occur. Previous studies have adequately described the risk of injury from jumps; these are among the most common movement elements leading to acute injuries [50].

It has been shown that the pointe shoe leads to a significantly increased metatarsal and ankle flexion after prolonged wear that causes increased strain and, therefore, a higher risk of injury [51]. Ballet dancers are also prone to the risk of ankle injuries due to possible differences in neuromuscular and, therefore, balance control [46]. Studies have already shown that the time required for stabilization in the anterior–posterior and medial–lateral directions after a vertical jump is longer in women than in men [46]. Based on other studies, the third phase of classical ballet training is the phase with the highest incidence of injury, despite a generally lower measurable and quantifiable ergonomic risk [50]. This risk is further exacerbated by the increasing training load due to rising ground reaction forces.

In view of the high ergonomic and kinematic demands placed on professional dancers in their daily training, as measured in the study, an adaptation of the training methodology seems appropriate (e.g., changing the order of the exercises, bringing forward the jumps, adagio and pirouettes to the end of the training session) with the aim of maintaining the effectiveness of the training and, at the same time, reducing the risk of developing musculoskeletal disorders. Consideration should also be given to gender-specific adaptation and weighting of the content, especially as anthropometric characteristics of the sexes can increase or reduce injury [2]. Women are generally smaller than men; thus, the female skeleton is around 25% lighter, and, as a result, stress fractures occur more frequently than in male dancers. The larger upper body of females in comparison to their overall height and their narrower shoulders and greater flexion in the hips also favor the valgus position in the knee joint and, thus, influence the forces acting on the knee and ankle joints. Furthermore, in female dancers, the extensors of the thigh are more pronounced than the flexors, which, in turn, puts more strain on the cruciate ligaments. In addition, women’s ligament structures are significantly more flexible and hormonally dependent, thus making them much more vulnerable to instability [2].

The effectiveness of measures to restructure the content of training to optimize load control and reduce ergonomic and injury-related risks requires further clarification in future studies. In particular, it should be investigated as to what extent all movement components of the phases must be performed unchanged on each training day, or whether a periodized training design is possible and sensible. In addition to dance training, the training plan should include cardiopulmonary components, such as interval training, as well as targeted strengthening exercises. The positive effects of such measures have already been proven in previous studies; however, these still require systematic evaluation [32,41]. Furthermore, it seems conceivable to integrate the regeneration phases systematically into the training plan and to revise the historically developed methodology critically by varying the static and joint-loading elements within the weekly training plan. A training modification could also include the adaptation and periodization of jump sequences, for example, by reducing the number of jumps or distributing the different types of jumps over several training days.

In addition to the ergonomic aspects of injury prevention, however, the working environment conditions should not be ignored as extrinsic factors (e.g., seasonal planning, ground conditions, light, climatic conditions) [52].

### Limitations of the Study

Rapid rotational movements of the head (e.g., pirouettes) and rapid foot movements (e.g., batterie, frappé) increased the risk of measurement inaccuracies with the risk of the fixation loosening. As a result, multiple measurements were necessary in individual cases (n = 3). The plantar flexion of the ankle and foot, which is characteristic of classical dance, could not be adequately mapped with the Xsens MVN Link, meaning that the results of mobility in the ankle joint may be falsified. This limited informative value was taken into account. Despite identical training specifications, the quality of the execution of technical dance movements does depend on numerous factors (e.g., physical prerequisites, anatomical conditions, motivation, and others). As a result, an influence on the individual measurement results cannot theoretically be ruled out. However, due to the professionalism and selection of the company members, it can be assumed that these are nuances that would not be statistically significant. At that, REBA risk categories are derived from factory workers. There is no evidence that REBA thresholds predict any dance injuries. High REBA scores may even reflect necessary athletic demands rather than poor design.

The ground reaction forces were only calculated indirectly using the 2nd derivative of the vertical pelvic position (acceleration of the pelvis). These results should, therefore, be interpreted with caution since shifts or inaccuracies cannot be ruled out. Further studies with force plates must contribute to the verification of the results. The Bonferroni–Holm correction was applied only between the individual comparisons and not across the full study. This might increase the risk of a false positive significance due to multiple comparisons. The Bonferroni–Holm correction is a very conservative estimation of multiple comparison effects. This is why the authors decided to use the Bonferroni correction only within the specific comparisons. Still, the chosen method increases the risk of false positive differences between genders, and the gender-specific differences should therefore not be overinterpreted. These significant findings might have low practical relevance due to overlapping ranges and the small sample size. Further limitations could be that REBA has so far not been validated for dance populations.

Despite all the limitations, this study provides the first key results that permit an assessment to be made based on the number of participants. However, further analyses or projects, possibly with larger groups, should follow in order to be able to make an even more differentiated classification of the risk of certain body regions.

## 5. Conclusions

Further studies are needed to validate the stresses and ergonomic risks faced by professional ballet dancers. This could involve combining multi-sensor measurement systems and longitudinal studies or intervention trials.

Despite the limitations mentioned above, this study can nevertheless be seen as a first step towards critically reflecting on traditional training methods and training content. This includes, for example, the variation of static and joint-stressing elements and the periodisation of jump sequences. The results contribute to raising awareness of the high level of work-related stress and problems, as well as existing and further recommendations for prevention and recognition of work-related musculoskeletal disorders in professional dance.

Translated with DeepL.com (free version).

## Figures and Tables

**Table 1:** Classification of the REBA score expressed as ergonomic risk [34].

REBA Score	Level of MSD Risk
1	negligible, no action required
2–3	low risk, change may be needed
4–7	medium risk, further investigation, change soon
8–10	high risk, investigate and implement change
11+	very high risk, implement change

**Table 2:** Sequence of the analysed exemplary training units of a condensed, typical classical dance training at a professional level. The terms listed in the first column equate to the generic terms (second column) and their variations. The phases were categorised in a modified form [3,6,36].

	Sequence of Movements, a Generic term for the Core Elements	Number of Cycles	Number of Executions for Weighted Calculations	Time of the Loads in Seconds
**Phase 1 barre:** Supported movement sequences were performed one after the other with the right or left leg as the free leg. High static component.				
1a.	Plié (right)	64 + 8 (balance)	1×	150 s
1b.	Plié (left)	64 + 8 (balance)	1×	150 s
2a.	Ronds de jambe à terre with port de bras (right)	36 + 16 (balance)	1×	135 s
2b.	Ronds de jambe à terre with port de bras (left)	36 + 16 (balance)	1×	135 s
3.	Battement frappé (right and left)	32 × 2 (right and left)	1×	25 s × 2
4.	Adagio with battement fondu, ronds de jambe en l’air, developpé (right and left)	32 × 2 (right and left)	1×	70 s × 2
5.	Grand battement with grand battement en cloche (right and left)	32 × 2 (right and left) + 8 (balance)	1×	37 s × 2
**Phase 2 centre:** Movement sequences in free space, which, due to their static component, were similar to the movement sequences at the barre (e.g., adagio) as well as pirouettes.				
6.	Port de bras and adagio (right and left)	32 × 2 (right and left)	1×	72 s × 2
7a.	Pirouette sequence (right) closed pirouettes and open pirouettes	32	3×	26 s × 3
7b.	Pirouette sequence I (left) see above	32	3×	26 s × 3
**Phase 3 jumps/allegro:** Jumps in place (petit allegro, allegro, grand allegro, batterie).				
8.	Warm-up (right and left)	16 × 2 (right and left)	1×	13 s × 2
9.	Jetés/glissades, jeté, assemblé u.a. (right and left)	16 × 2 (right and left)	3×	12 s × 2 × 3
10.	Batterie combination (right and left)	16 × 2 (right and left)	2×	13 s × 2 × 2
11a.	Grand allegro (z. B. Sissonnes, grand jeté en tournant) (right)	16	4×	17 s × 4
11b.	Grand allegro (z. B. Sissonnes, grand jeté en tournant) (left)	16	4×	17 s × 4
12.	Warm-up (pointe shoes) right and left	16 × 2 (right and left)	1×	16 s × 2
13a u. b.	En mange pirouettes (f) demi pointe (right + left, a) and pointe (right, b)	8 × 2 (right and left)	2×	10 s × 3
14	Grand allegro en manege (m)	8	1×	10 s
**Total duration of training**				**1358 s (m)** **1410 s (f)**

**Table 3:** Specific adjustment parameters for calculating the REBA score.

Steps	Parameters	Defined Movement According to the REBA Worksheet	Implemented in MATLAB
1	Movement of the neck (flexion and extension)	Forward head tilt - 0–20° ⇨ +1 - >20° ⇨ +2 - <0° ⇨ +2	- Joint name: “jC1Head” - Direction of movement: flexion, limits are taken exactly from the REBA worksheet.
1a	Rotation of the head, lateral tilt of the head	- Head rotation ⇨ +1 - Head tilt ⇨ +1	- Joint name: “jC1Head” - Direction of movement: rotation, limits: <−10° and >+10° ⇨ +1 - Direction of movement: flexion, limits: <−10° and >+10° ⇨ +1
2	Movement of the trunk (flexion/extension)	The movement of the trunk is also assessed in terms of the degree of flexion, extension and rotation. - Extension 0° ⇨ +1 - Extension 0–20° ⇨ +2 - Flexion 0–20° ⇨ +2 - Extension > 20° ⇨ +3 - Flexion 20–60° ⇨ +3 - Flexion > 60° ⇨ +4	- For the trunk the joints “jL5S1”, “jL4L3”, “jL1ST12”, “jT1C7” are added. - Direction of movement: flexion, limits are taken exactly from the REBA worksheet.
2a		Deviation of the trunk from - The sagittal plane ⇨ +1 - Side bending ⇨ +1	- For the trunk the joints “jL5S1”, “jL4L3”, “jL1ST12”, “jT1C7” are added. - Direction of movement: rotation, limits: <−10° and >+10° ⇨ +1 - Direction of movement: flexion, limits: <−10° and >+10° ⇨ +1
3	Movement of the leg	- Both legs extended on the floor ⇨ +1 - One leg raised ⇨ +2 - Legs bent at 30–60° ⇨+1 - Legs bent at >60° ⇨ +2	The ‘footContact’ parameter is used to determine whether neither leg, one leg or both legs are in contact with the ground. - Knee flexion - Joint name: “jLeftKnee” and “jRightKnee” - Direction of movement: flexion - The REBA criteria are first calculated separately for both sides and then combined (maximum value from [left; right])
4	Look up the posture score for the upper body and legs	REBA Table A	Similar to REBA worksheet
5	Force and load	Force evaluation - Load < 11 lbs. ⇨ 0 - Load 11 to 22 lbs. ⇨ +1 - Load > 22 lbs. ⇨ +2 Fast load acceleration - additionally ⇨ +1	- No additional weight is carried when dancing ⇨ elimination of load. - The forces during take-off and landing are taken into account. Vertical pelvic acceleration is used. >5 BW ⇨ +1 >10 BW ⇨ +2
6	Addition of 4 and 5		Similar to REBA worksheet
7	Movement of the upper arm (flexion/extension)	- Upper arm position neutral −20°–+20° ⇨ +1 - Upper arm position in extension −20° ⇨ +2 - Upper arm position in flexion 20–45° ⇨ +2 - Upper arm position in flexion 45–90° ⇨ +3 - Upper arm position in flexion 90° ⇨ +4	- Joint name: “jLeftShoulder” and “jRightShoulder” - Direction of movement: flexion, limits are taken exactly from the REBA worksheet. - Direction of movement: abduction, limits are taken exactly from the REBA worksheet.
7a	Movement of the upper arm with additional rotation, abduction or support	- Additional rotation of the shoulder ⇨ +1 - Additional abduction ⇨ +1 - Upper arm is supported or rested ⇨ −1	- Joint name: “jLeftT4Shoulder” and “jRightT4Shoulder” - Direction of movement: flexion, limits: >10° ⇨ +1 Support from the upper arm is not taken into account.
8	Movement of the forearm	- Forearm flexion 60–100° ⇨ +1 - Forearm flexion 0–60° ⇨ +2 - Forearm flexion > 100° ⇨ +2	- Joint name: “jLeftElbow” and “jRightElbow” - Direction of movement: flexion, limits are taken exactly from the REBA worksheet.
9	Movement of the wrist	- Wrist in neutral position −15–+15° ⇨ +1 - Wrist in flexion > 15° ⇨ +2 - Wrist in extension > 15° ⇨ +2	- Joint name: “jLeftMWUist” and “jRightMWUist” - Direction of movement: flexion, limits are taken exactly from the REBA worksheet.
9a	Rotation of the wrist	The wrist score is supplemented if the joint bends or twists away from the midline ⇨ +1	- Joint name: “jLeftMWUist” and “jRightMWUist” - Direction of movement: rotation <−10° ⇨ +1 >+10° ⇨ +1 - Joint name: “jLeftMWUist” and “jRightMWUist” - Direction of movement: abduction <−45° ⇨ +1 >+45° ⇨ +1
10	Looking up the posture score for arms	REBA Table B	Similar to REBA worksheet
11	Evaluation of the handle (coupling value)	Add coupling point score - Well-fitting grip and medium strength grip ⇨ +0 - Acceptable but not ideal grip or coupling with another acceptable body part ⇨ +1 - Hand grip not acceptable, but possible ⇨ +2 - No grip, unfavourable, unsafe with other body part, unacceptable ⇨ +3	Not taken into account, as no devices are held.
12	Looking up the posture assessment	REBA Table C	Similar to REBA worksheet
13	Activity scores	Activity scores—these take into account - the duration of the movement ⇨ +1 - the repetition ⇨ +1 range of motion ⇨ +1	- Static: no more than 7.5° difference over 10 s ⇨ +1 - Dynamic: an average power frequency of >5 ⇨ +1
REBA score		Sum of 12 and 13	Similar to REBA worksheet

**Table 4:** Gender-specific differences in overall training according to the REBA scores (n = 28). Significant values are shown in bold. The first column is the REBA score, the second column is the Wilcoxon–Mann–Whitney U test score and the statistical significance. Note that due to the multiple comparisons, a Bonferroni–Holm correction has to be applied. The 3rd column states the Cliff’s delta δ effect size. REBA scores of 6 and 7 showed a large effect size and can therefore be considered as a meaningful difference between genders.

REBA Score	Wilcoxon–Mann–Whitney U Test (Bonferroni–Holm Corrected Limit: 0.0042)	Cliff’s Delta δ Effect Size
1	MWU = −1.484 (*p* = 0.138)	−0.333
2	MWU = 0.731 (*p* = 0.465)	0.167
3	MWU = 2.148 (*p* = 0.032)	0.480
4	MWU = 1.173 (*p* = 0.241)	0.265
5	MWU = 0.863 (*p* = 0.388)	0.196
6	MWU = −2.989 (*p* = 0.003)	−0.667
7	MWU = −0.066 (*p* = 0.947)	−0.020
8	MWU = 0.155 (*p* = 0.877)	0.039
9	MWU = −3.343 (*p* = 0.001)	−0.745
10	MWU = −2.236 (*p* = 0.025)	−0.500
11	MWU = −1.749 (*p* = 0.080)	−0.333
12	MWU = −0.770 (*p* = 0.441)	−0.059

**Table 5:** Phase-specific gender differences in the individual REBA scores (n = 28). The first column is the REBA score, the second column is the Wilcoxon–Mann–Whitney U test score (MWU) and the statistical significance (*p* < 0.05 significant). Note that due to the multiple comparisons, a Bonferroni–Holm correction has to be applied. Significant values are shown in bold. The 3rd column reports the Cliff’s delta (δ) effect size. REBA score 3 for phase III; REBA score 6 for phase II and III; REBA score 9 for phase I and II, and REBA score 10 for phase II had large effect sizes.

REBA Score	Phase 1		Phase 2		Phase 3	
	Wilcoxon Mann–Whitney U Test (Bonferroni–Holm korrigiertes Limit: 0.0042)					
	MWU test	Cliff’s delta δ		Cliff’s delta δ		Cliff’s delta δ
1	MWU = −0.673 (*p* = 0.501)	0.156	MWU = 1.339 (*p* = 0.180)	−0.305	MWU = 0.243 (*p* = 0.808)	−0.042
2	MWU = −2.577 (*p* = 0.010)	0.583	MWU = 0.282 (*p* = 0.778)	−0.070	MWU = −0.766 (*p* = 0.444)	0.177
3	MWU = −1.787 (*p* = 0.074)	0.406	MWU = −1.129 (*p* = 0.259)	0.262	MWU = −2.252 (*p* = 0.024)	0.510
4	MWU = −1.741 (*p* = 0.082)	0.396	MWU = 1.411 (*p* = 0.158)	−0.326	MWU = −1.230 (*p* = 0.219)	0.281
5	MWU = −0.998 (*p* = 0.318)	0.229	MWU = 0.988 (*p* = 0.323)	−0.230	MWU = −0.859 (*p* = 0.390)	0.198
6	MWU = 1.834 (*p* = 0.067)	−0.417	**MWU = 3.857 (*p* < 0.001)**	−0.882	**MWU = −3.923 (*p* = 0.000)**	0.885
7	MWU = −0.070 (*p* = 0.944)	0.021	MWU = 0.612 (*p* = 0.541)	−0.144	MWU = −1.323 (*p* = 0.186)	0.302
8	MWU = −0.766 (*p* = 0.444)	0.177	MWU = 0.941 (*p* = 0.347)	−0.219	MWU = −1.137 (*p* = 0.255)	0.260
9	MWU = 2.762 (*p* = 0.006)	−0.625	**MWU = 3.434 (*p* < 0.001)**	−0.786	MWU = 1.648 (*p* = 0.099)	−0.375
10	MWU = 1.787 (*p* = 0.074)	−0.406	MWU = 2.540 (*p* = 0.011)	−0.583	MWU = 0.070 (*p* = 0.944)	−0.021
11	MWU = 1.539 (*p* = 0.124)	−0.292	MWU = 0.731 (*p* = 0.465)	−0.059	No loadings	
12	No loadings		MWU = 0.731 (*p* = 0.465)	−0.059	No loadings	

**Table 6:** Pairwise comparisons of the three phases for REBA and load. All three phases were significantly different from each other for both the REBA scores and within the loads. The mutual comparisons for the REBA score are presented in the upper section of the table, and the mutual comparisons for the three different phases for the load measure are presented in the lower section.

REBA Scores					
Group Comparison A, B		Lower Limit	A-B	Upper Limit	*p*-Value
Phase 1	Phase 2	−35.30	−19.70	−4.10	**0.007**
Phase 1	Phase 3	7.60	23.20	38.90	**0.001**
Phase 2	Phase 3	27.40	43.00	58.60	<**0.001**
Load					
Group Comparison A, B		Lower Limit	A-B	Upper Limit	*p*-Value
Phase 1	Phase 2	−38.30	−22.70	−7.10	**0.001**
Phase 1	Phase 3	−67.50	−51.90	−36.30	<**0.001**
Phase 2	Phase 3	−44.80	−29.10	−13.50	<**0.001**

**Table 7:** Gender-specific representation of the ergonomic risk based on the REBA score in the individual movement sequences in the respective phases 1–3, in % (n = 28). MWU = Wilcoxon–Maann–Whitney U test; *p* = probability (*p* < 0.05 significant). The REBA scores in columns 3 to 7 are cumulated within the respective ranges based on the risk bins from the REBA assessment. The presented values are the relative occurrence of the REBA score for the specific bin for male (upper line) and female (lower line) ballet dancers. The average REBA score is presented in column 8 followed by the Wilcoxon–Mann–Whitney U test (upper line) and the respective difference for the gender difference. Note that a multiple comparison correction has to be applied => 0.003 is the upper threshold for a significant difference. The last column presents the cliff’s delta δ as an estimate of the effect size. Plié; Adagio with battement fondu, ronds de jambe en l’air, developpé and Port de bras and Adagio showed large effect sizes.

Phase	Training Content	REBA 1	REBA 2–3	REBA 4–7	REBA 8–10	REBA 11	Average REBA Score	MWU (p)	Cliff’s Delta δ
Phase 1	Plié	1% (m) 0% (w)	7% (m) 4% (w)	61% (m) 60% (w)	31% (m) 36% (w)	0%	6.33 (m) 6.70 (w)	−3.17 (0.002)	−0.513
	Ronds de jambe à terre with port de bras	1% (m) 0% (w)	12% (m) 8% (w)	66% (m) 68% (w)	21% (m) 24% (w)	0%	5.63 (m) 6.11 (w)	−2.34 (0.02)	−0.370
	Battement frappé	0%	7% (m) 5% (w)	84% (m) 85% (w)	9% (m) 10%(w)	0%	5.25 (m) 5.49 (w)	−0.86 (0.39)	−0.198
	Adagio with battement fondu, ronds de jambe en l’air, developpé	0%	10% (m) 5% (w)	58% (m) 55% (w)	32% (m) 40% (w)	0%	6.56 (m) 7.04 (w)	−3.10 (0.002)	−0.733
	Grand battement with grand battement en cloche	0%	4% (m) 3% (w)	70% (m) 70% (w)	26% (m) 27% (w)	0%	6.22 (m) 6.33 (w)	−1.20 (0.23)	−0.285
Phase 2	Port de bras and Adagio	1% (m) 0% (w)	10% (m) 5% (w)	50% (m) 55% (w)	39% (m) 40% (w)	0%	6.24 (m) 6.75 (w)	−3.52 (<0.001)	−0.784
	Pirouette sequence (right) closed pirouettes and open	0%	10% (m) 5% (w)	63% (m) 63% (w)	28% (m) 32% (w)	0%	6.39 (m) 6.44 (w)	−1.79 (0.07)	−0.291
	Warm up (point shoes) right and left	1%	11% (w)	71% (w)	17% (w)	0%	not applicable	not applicable	
Phase 3	Warm-up jumps	0%	27% (m) 20% (w)	70% (m) 77% (w)	3% (m) 3% (w)	0%	4.38 (m) 4.30 (w)	−0.53 (0.59)	−0.125
	Jetés/glissades, jeté, assemblé etc.	0% (m) 1% (w)	20% (m) 12% (w)	69% (m) 68% (w)	11% (m) 19% (w)	0%	5.17 (m) 5.52 (w)	−2.13 (0.03)	−0.471
	Batterie combination	0%	12% (m) 8% (w)	71% (m) 70% (w)	17% (m) 23% (w)	0%	5.46 (m) 5.93 (w)	−1.37 (0.17)	−0.312
	Grand allegro (e.g., Sissonnes, grand jeté en tournant)	1% (m) 3% (w)	11% (m) 9% (w)	58% (m) 58% (w)	30% (m) 30% (w)	0%	6.3 (m) 6.15 (w)	0.25 (0.81)	0.048
	En manege (m) Piques en diagonale (w)	0% 0%	10% 5%	76% 72%	14% 23%	0% 0%	not applicable not applicable	not applicable not applicable	

## Data Availability

Data is provided within the manuscript.

## Supplementary Materials

The REBA source code can be accessed through the GitHub repository: https://github.com/ChristianMaurerGrubinger/2025-REBA (Accessed on 1 November 2025).
